# Bisphenol-A (BPA) in Foods commonly consumed in Southwest Nigeria and its Human Health Risk

**DOI:** 10.1038/s41598-019-53790-2

**Published:** 2019-11-25

**Authors:** Adebola A. Adeyi, Babafemi A. Babalola

**Affiliations:** 1 Department of Chemistry, University of Ibadan, Ibadan, Oyo State Nigeria; 2 Basel Convention Coordinating Centre for Training and Technology Transfer for Africa Region, University of Ibadan, Ibadan, Oyo State Nigeria

**Keywords:** Mass spectrometry, Environmental monitoring

## Abstract

Bisphenol-A (BPA) is a synthetic chemical ubiquitous in the environment and listed as an endocrine disruptor. It has the tendency of migrating into food stored in materials containing it. This study, therefore, determines the concentrations of BPA in foods commonly consumed in Southwest Nigeria by the adult population and also estimates the risk associated with human exposure. Eight different food categories were selected for this study. Standard QuEChERS protocol was used for sample extraction and analysed using gas chromatography-mass spectrometry (GC-MS). Vegetable oil had the highest BPA concentration (28.4 ng/g). This was followed by aquatic canned fish (26.3 ng/g), canned beef (21.3 ng/g) and crayfish (17.5 ng/g). These concentrations were below the 600 ng/g limit of the European Commission for BPA in foods. Bisphenol-A was not detected in raw beef, chicken, cheese, apple, tomatoes, beans and rice; and chicken eggs. The adult population had an average dietary intake of 30.4 ng/kg bw/day. There is no likely occurrence of harmful health effects of BPA in the selected foods with respect to the current concentrations found therein. However, routine monitoring is recommended to prevent human exposure to BPA.

## Introduction

Bisphenol-A, 4, 4′-dihydroxy-2, 2-diphenylpropane, is a synthetic organic compound obtained from the condensation of acetone and phenol^1^ and a high volume industrial chemical. It has drawn global attention in recent years^2^ due to its ability to interfere with the functioning of endocrine systems. It is widely used in the production of polycarbonates in the manufacture of food storage containers including feeding and non-returnable bottles and various kitchen items; epoxy resins^3–6^ used in the production of lacquers, the inner coating of food cans and thermal papers^7–10^; personal care products including sunscreen lotions, facial lotions and cleanser, nail polish^8,11^ and toys^12^. Plastic materials and articles intended for food^6,13^ storage can release BPA during production, handling, packaging and transporting.

Bisphenol-A (BPA) belongs to category 1 of Endocrine Disruptive Chemicals (EDCs) that is acutely toxic to living organisms^11,14^ in relation to its clear evidence of endocrine disrupting activity^15^. Recent animal studies revealed that exposure to BPA led to obesity, thyroid dysfunction, and cardiovascular diseases^16^. BPA exposure has been reported to cause type 2 diabetes, leptin levels in pregnant mice and their offspring^17^, and interfered with the glucose and lipid homeostasis in female mice and their offspring^18^. Fetal reproductive system development in humans has been linked to maternal exposure to BPA^19^. Adverse effects of BPA on male reproductive function^20–22^, disruption of thyroid function^23^, metabolic syndrome such as hypertension, insulin resistance, diabetes mellitus, and obesity^24,25^, and cardiovascular diseases^26^ have been reported. In 2013, it was reported that exposure of children of ages 3, 5 and 7 to higher concentrations of BPA can lead to asthma development in the later years^27^.

Humans are exposed to BPA via different sources which include water, effluent, air, dust and food^28–33^. However, diet is a critical route of BPA exposure by ingestion of water and food^34^ that are contaminated. Bisphenol-A gets into foods mainly through leaching from the lining of beverage and food cans^35^. Foods are contaminated with BPA probably in the course of production, handling, packaging, and transportation^36^. Exposure to BPA has been detected in low concentrations in non-food sources than that of food^37^. The Consumer Reports magazine reported the level of BPA higher than the Food and Drug Administration ‘Cumulative Exposure Daily Intake’ limit when it was determined in some canned foods and beverages^38^. Research has also linked drinking from polycarbonate bottles to increased urinary BPA concentration^39^.

To ensure the safety of food contact materials, the European Union has established restrictions of use and migration limit of 0.6 mg BPA per kg food^40^. However, it was prohibited in the manufacturing of plastic for infant feeding bottles since 1 May 2011 (Commission Implementing Regulation (EU) No. 321/2011). The European Food Safety Authority has established the maximum acceptable level of BPA at 4 µg/kg-bw/day^41^. Also, Health Canada has established the tolerable daily intakes (TDI) for BPA at 0.025 mg/kg-bw/day^42^.

The advantages offered by QuEChERS (Quick, Easy, Cheap, Effective, Rugged, and Safe) technique over the traditional methods of food analysis, which is laborious, time consuming, and requires the use of large volume of organic solvent, have been reported^43,44^. QuEChERS is a simple, rapid, and cost effective sample preparation method^43,44^. This study assesses the levels of BPA in foods commonly consumed in Southwest Nigeria using QuEChERS protocol prior to derivatisation of the extracts and analyses by GC-MS and also evaluates the health risk of BPA exposure from food intake by adult population in Southwest Nigeria.

## Results

### Levels of BPA in selected foods

The levels of BPA in the selected food samples collected in Lagos and Ibadan varied greatly. Table 1 shows the concentrations of BPA in the selected foods commonly consumed in Southwest Nigeria. There were variations in the concentration of BPA in the food categories and by food types. By food categories, the mean concentrations of BPA in the foods ranged from ND (raw beef and chicken) - 21.3 ng/g (canned beef) in the meat products; 0.78 (frozen fish)-26.3 ng/g (canned fish) in the aquatic foods; ND (raw cheese)-4.8 ng/g (canned evaporated milk) in the dairy foods; 0.41–28.4 ng/g (vegetable oils) in the edible oils; ND (chicken eggs); ND (raw apple)-0.58 ng/g (processed apple juice) in fruits; ND (raw tomato)- 5.66 ng/g (canned tomato paste) in the vegetables; and ND (beans and rice) in the cereals categories, respectively. The mean concentrations (ng/g), standard deviations and median of BPA in the selected food categories were 4.28 ± 6.0 (2.21 ng/g) in the meat products, 7.56 ± 3.1 (7.49 ng/g) in the aquatic foods, 1.42 ± 1.3 (1.89 ng/g) in dairy products, 6.39 ± 0.4 (6.39 ng/g) in edible oils, ND in chicken eggs, 0.20 ± 0.3 (0.20 ng/g) in fruits, 1.11 ± 1.6 (1.11 ng/g) in vegetables, and ND in cereals (Supplementary Table S1). The order of BPA concentrations in the food categories were aquatic foods > edible oils > meat products > dairy products > vegetables > fruits > chicken eggs = cereals (Supplementary Table S1).

**Table 1:** Concentrations (ng/g) of BPA in selected foods commonly consumed by adults’ in Southwest Nigeria.

Food categories	Food types	Number of samples (n)	Concentrations of BPA (ng/g)
Meat products	Raw Beef 1	3	ND
	Raw Beef 2	3	ND
	Raw Beef 3	3	ND
	Raw Beef 4	3	ND
	Raw Beef 5	3	ND
	Raw Beef 6	3	ND
	Mean		—
	Stdev		—
	Min		—
	Max		—
	Median		—
	Raw Chicken 1	3	ND
	Raw Chicken 2	3	ND
	Raw Chicken 3	3	ND
	Raw Chicken 4	3	ND
	Raw Chicken 5	3	ND
	Raw Chicken 6	3	ND
	Raw Chicken 7	3	ND
	Raw Chicken 8	3	ND
	Mean		—
	Stdev		—
	Min		—
	Max		—
	Median		—
	Canned Beef Exeter1	1	5.88
	Canned Beef Costa 1	1	21.3
	Canned Beef Exeter 2	1	6.47
	Canned Beef Costa 2	1	17.2
	Mean		12.7
	Stdev		7.7
	Min		5.88
	Max		21.3
	Median		11.9
	Canned chicken ZWAN 1	1	6.36
	Canned chicken AlTaziah 1	1	2.94
	Canned chicken ZWAN 2	1	3.77
	Canned chicken AlTaziah 2	1	4.63
	Mean		4.42
	Stdev		1.5
	Min		2.94
	Max		6.36
	Median		4.20
Aquatic foods	Frozen fish 1	3	6.04
	Frozen fish 2	3	2.46
	Frozen fish 3	3	3.16
	Frozen fish 4	3	7.85
	Frozen fish 5	3	4.06
	Frozen fish 6	3	0.78
	Mean		4.06
	Stdev		2.5
	Min		0.78
	Max		7.85
	Median		3.61
	Dried fish 1	3	7.36
	Dried fish 2	3	7.09
	Dried fish 3	3	5.01
	Dried fish 4	3	6.40
Aquatic foods	Dried fish 5	3	9.40
	Dried fish 6	3	2.28
	Mean		6.26
	Stdev		2.4
	Min		2.28
	Max		9.40
	Median		6.75
	Canned fish Flake Tuna 1	1	2.91
	Canned fish Costa Mackerel 1	1	26.3
	Canned fish Flake Tuna 2	1	1.66
	Canned fish Costa Mackerel 2	1	13.9
	Mean		11.2
	Stdev		11
	Min		1.66
	Max		26.3
	Median		8.41
	Crayfish 1	3	13.0
	Crayfish 2	3	7.20
	Crayfish 3	3	9.90
	Crayfish 4	3	11.8
	Crayfish 5	3	17.5
	Crayfish 6	3	1.20
	Crayfish 7	3	2.66
	Crayfish 8	3	6.49
	Mean		8.72
	Stdev		5.4
	Min		1.20
	Max		17.5
	Median		8.55
Dairy foods	Raw cheese 1	1	ND
	Raw cheese 2	1	ND
	Raw cheese 3	1	ND
	Raw cheese 4	1	ND
	Mean		—
	Stdev		—
	Min		—
	Max		—
	Median		—
	Processed cheese- Happy cow 1	1	1.78
	Processed cheese- President Alvita 1	1	2.82
	Processed cheese- Happy cow 2	1	1.45
	Processed cheese- President Alvita 2	1	3.43
	Mean		2.37
	Stdev		0.9
	Min		1.45
	Max		3.43
	Median		2.30
	Evaporated milk- Hollandia 1	1	0.73
	Evaporatec milk- Three crowns 1	1	4.80
	Evaporated milk- Hollandia 2	1	0.20
	Evaporatec milk- Three crowns 2	1	1.86
	Mean		1.89
	Stdev		2.1
	Min		0.20
	Max		4.80
	Median		1.29
Edible oils	Vegetable oil 1	1	8.23
	Vegetable oil 2	1	5.21
	Vegetable oils 3	3	15.7
	Vegetable oils 4	3	1.30
	Vegetable oils 5	3	0.85
	Vegetable oil 6	1	28.4
	Vegetable oil 7	1	4.23
	Vegetable oils 8	3	1.87
	Vegetable oils 9	3	0.47
	Vegetable oils 10	3	0.41
	Mean		6.67
	Stdev		9.0
	Min		0.41
	Max		28.4
	Median		3.05
	Palm oils 1	3	5.41
	Palm oils 2	3	9.82
	Palm oils 3	3	11.6
	Palm oils 4	3	4.65
	Palm oils 5	3	2.83
	Palm oils 6	3	2.35
	Mean		6.11
	Stdev		3.8
	Min		2.35
	Max		11.6
	Median		5.03
Chicken eggs	Chicken eggs 1	3	ND
	Chicken eggs 2	3	ND
	Chicken eggs 3	3	ND
	Chicken eggs 4	3	ND
	Chicken eggs 5	3	ND
	Chicken eggs 6	3	ND
	Mean		—
	Stdev		—
	Min		—
	Max		—
	Median		—
Fruits	Raw Apples 1	3	ND
	Raw Apples 2	3	ND
	Raw Apples 3	3	ND
	Raw Apples 4	3	ND
	Mean		—
	Stdev		—
	Min		—
	Max		—
	Median		—
	Processed apple juice-Chivita 1	1	0.36
	Processed apple juice-Happy hour 1	1	0.31
	Processed apple juice-Chivita 2	1	0.58
	Processed apple juice-Happy hour 2	1	0.36
	Mean		0.40
	Stdev		0.1
	Min		0.31
	Max		0.58
	Median		0.36
Vegetables	Raw Tomato 1	3	ND
	Raw Tomato 2	3	ND
	Raw Tomato 3	3	ND
	Raw Tomato 4	3	ND
	Mean		—
	Stdev		—
	Min		—
	Max		—
	Median		—
	Canned tomato paste Redsarsa 1	1	0.42
	Canned tomato paste Gino 1	1	0.81
	Canned tomato paste Redsarsa 2	1	5.66
	Canned tomato paste Gino 2	1	1.96
	Mean		2.21
	Stdev		2.4
	Min		0.42
	Max		5.66
	Median		1.38
Cereals	Beans 1	3	ND
	Beans 2	3	ND
	Beans 3	3	ND
	Beans 4	3	ND
	Beans 5	3	ND
	Beans 6	3	ND
	Mean		—
	Stdev		—
	Min		—
	Max		—
	Median		—
	Rice 1	3	ND
	Rice 2	3	ND
	Rice 3	3	ND
	Rice 4	3	ND
	Rice 5	3	ND
	Rice 6	3	ND
	Mean		—
	Stdev		—
	Min		—
	Max		—
	Median		—

By food types, BPA was not detected in raw beef, raw chicken, raw cheese, raw tomato, chicken eggs, raw apples, beans and rice (Table 1). The concentrations of BPA by food types ranged from 5.88–21.3 ng/g in canned beef, 2.94–6.36 ng/g in canned chicken, 0.78–7.85 ng/g in frozen fish, 2.28–9.40 ng/g in dried fish, 1.66–26.3 ng/g in canned fish, 1.20–17.5 ng/g in crayfish, 1.45–3.43 ng/g in processed cheese, 0.20–4.80 ng/g in canned evaporated milk, 0.41–28.4 ng/g in vegetable oil, 2.35–11.6 ng/g in palm oil, 0.31–0.58 ng/g in processed apple juice, and 0.42–5.66 ng/g in canned tomato paste. However, it was observed that foods packaged in lined cans and plastic bags had the highest concentrations of BPA. This might be as a result of leaching of BPA from the packaging materials into the foods stored therein. In the meat products category, canned beef had the highest mean concentration (12.7 ± 7.7 ng/g) of BPA (Table 1). This was followed by canned chicken (4.42 ± 1.5 ng/g) while BPA was not detected in raw beef and chicken. In the aquatic foods category, canned fish had the highest concentrations (11.2 ± 11 ng/g), followed by crayfish, dried fish and frozen fish. Their concentrations were 8.72 ± 5.4 ng/g, 6.26 ± 2.4 ng/g and 4.06 ± 2.5 ng/g, respectively. The concentrations in the dairy products category was 2.37 ± 0.9 ng/g in the processed cheese, 1.89 ± 2.1 ng/g in the canned evaporated milk and not detected in the raw cheese. In the edible oils category, vegetable oil had the highest concentration, 6.67 ± 9.0 ng/g, while it was 6.11 ± 3.8 ng/g in the palm oil. BPA was not detected in chicken eggs, beans and rice. Processed apple juice in the fruit category had the highest mean concentration, 0.40 ± 0.1 ng/g, while BPA was not detected in raw apples. In the vegetable category, canned tomato paste had the highest BPA concentration (2.21 ± 2.4 ng/g) while it was not detected in raw tomatoes. BPA was not detected in all the raw foods and chicken eggs considered in this study but was in varied concentrations in the packaged foods and the aquatic food products. The presence of BPA in the aquatic foods might be associated with the fact that BPA was a high volume chemical used in the production of polycarbonates and epoxy resin. Also, aquatic environment is the final sink of all environmental contaminants including BPA from plastic materials which is recently of an environmental concern.

### Results of statistical analysis

One way analysis of variance (ANOVA) of the concentrations of BPA in foods commonly consumed in Southwest Nigeria showed a significant variation within the food types and across the food categories (Supplementary Table S2). The post-hoc homogenous subset (Duncan) of BPA in foods revealed that the concentrations of BPA in some food types such as canned beef, canned fish and crayfish were higher and different from other food types (Supplementary Table S3).

### Results of human health risk assessment

#### Results of food consumption survey

A total of 250 households, which comprised of 100 males and 150 females, participated in this study out of the 400 households surveyed across the 10 LGAs in Lagos and Ibadan. This number was based on the total number of respondents which volunteered to participate in the study and were all healthy adult without any form of physical deformity and sickness. Only one person per household was permitted to participate in this study. The questionnaire administered is presented in Supplementary Table S4. Forty five respondents were between the ages of 26 and 35, 51 respondents were between the ages of 36 and 45, 60 respondents were between the ages of 46 and 55, 62 respondents were between the ages of 56 and 65, while 32 respondents were over 66 years. The average weights of both adults’ men and women were 63 and 67 kg, respectively while 65 kg was the estimated average body weights (BW) of an adult in Southwest Nigeria. The average heights of women and men were 1.32 m and 1.55 m, respectively. Ninety per cent of the respondents were from the Yoruba ethnic group while the remaining 10% were from the Igbo ethnic group. Based on marital status, 98% of the respondents were married while only 2% were not currently married. According to their educational levels, 85% of the respondents had tertiary education while 15% had secondary school education (Supplementary Table S5).

The frequency of food consumption and the ingestion rate (in g/day) of each food types are presented in Table 2. In most cases, the raw foods were consumed 2–3 times per week while the canned foods were consumed only 1–3 times per month. The average ingestion rates (in g/day) of the food categories by female respondents was 80.8, 60.5, 59, 83.5, 35, 86, 69, and 175; and male respondents was 64.3, 59.5, 51, 76.5, 25, 74, 51, and 165 of aquatic foods, meat products, dairy products, edible oils, chicken eggs, fruits, vegetables and cereals, respectively. The estimated average ingestion rates of the food categories were higher for female respondents than for the males. In most cases, the females consumed more of the food types than the male respondents except raw beef and beans  which the males consumed more than the females. The data obtained was subjected to statistical T- test distribution to know if there are significant differences between the average ingestion rates by sex. There is a significant difference between the average ingestion rates by sex (that is males and females) in Southwest Nigeria (p = 0.0013 at α = 0.05).

**Table 2:** Frequency of consumption and estimated ingestion rate (g/day) of selected foods commonly consumed in Southwest Nigeria by sex.

Food categories	Food types	Average frequency of consumption	Average ingestion rate by females	Average ingestion rate by males
Meat products	Raw beef	2–3 times per week	110	130
	Raw chicken	2–3 times per week	110	90
	Canned beef	1–3 times per month	13	11
	Canned chicken	1–3 times per month	9	7
Average			60.5	59.5
Aquatic foods	Frozen fish	2–3 times per week	160	140
	Dried fish	2–3 times per week	120	80
	Canned fish	1–3 times per month	23	17
	Crayfish	2–3 times per week	20	20
Average			80.8	64.3
Dairy products	Raw cheese	2–3 times per week	60	40
	Processed packaged cheese	1–3 times per month	7	3
	Evaporated milk	1–3 times per month	110	110
Average			59	51
Edible oils	Vegetable oil	2–3 times per week	86	74
	Palm oil	2–3 times per week	81	79
Average			83.5	76.5
Chicken eggs	Eggs	2–3 times per week	35	25
Fruits	Raw apple	2–3 times per week	22	18
	Processed apple juice	1–3 times per month	150	130
Average			86	74
Vegetables	Raw tomato	2–3 times per week	83	57
	Canned tomato	1–3 times per month	55	45
Average			69	51
Cereals	Beans	2–3 times per week	190	210
	Rice	2–3 times per week	160	120
Average			175	165

#### Estimated daily intake and health risk index (HI) of BPA in selected foods commonly consumed in Southwest, Nigeria

**Figure 1:**
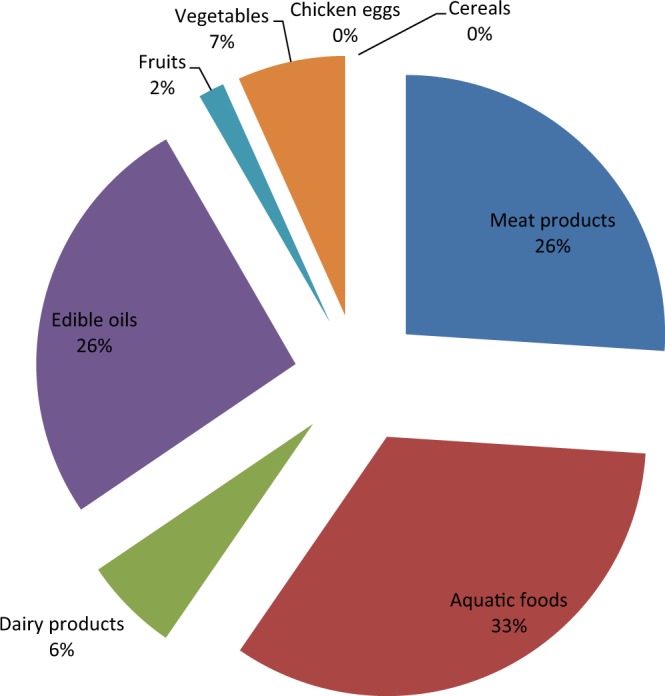
Contribution of selected food categories commonly consumed in Southwest Nigeria to daily intake of BPA.

Variations in the percentage daily intake of BPA in foods commonly consumed in Southwest Nigeria are shown in Fig. 1. Aquatic foods category had the highest contribution (33%) to the total daily intake of BPA while chicken eggs and cereal did not contribute to the total daily intake of BPA in Southwest Nigeria. The order of estimated daily intake of BPA was: aquatic foods (33%) > edible oils (26%) = meat products (26%) > vegetables (7%) > dairy products (6%) > fruits (2%) > chicken eggs (0%) = cereals (%). The estimated daily intake of BPA in the selected food categories are presented in Table 3. The daily intake of BPA in meat products, aquatic foods, dairy products, edible oils, fruits, vegetable, chicken eggs, and cereals were 7.91, 10.2, 1.80, 7.95, 0.49, 2.04, 0.00 and 0.00 ng/kg/day, respectively. The total daily intake of BPA in the selected food categories was 30.4 ng/kg/day. By food types, the estimated daily intake of BPA ranged from 0.00–9.58 ng/kg/day (Table 4). The highest estimated daily intake was obtained in dried fish, followed by frozen fish, vegetable oils, palm oils, canned fish, canned evaporated milk, crayfish, canned beef, canned tomatoes, apple juice, canned chicken, and processed cheese. Raw beef, raw chicken, raw cheese, chicken eggs, raw apples, raw tomatoes, beans and rice did not contribute to the estimated total daily intake of BPA in Southwest Nigeria. The highest estimated daily intake of BPA was in dried fish, followed by frozen fish, canned fish and crayfish in the aquatic food category (Table 4). In the meat products category, the highest estimated daily intake of BPA was in canned beef and canned chicken while BPA was not ingested in raw beef and raw chicken, respectively. In dairy products category, the highest daily intake of BPA was in canned evaporated milk, followed by packaged cheese while raw cheese did not contribute to the total estimated daily intake. In the edible oils category, considerable amount of BPA was ingested in the vegetable and palm oils while chicken eggs contributed nothing to the estimated daily intake of BPA. In the fruits and vegetables categories, raw apples and tomatoes, respectively did not contribute to the estimated daily intake of BPA while apple juice and canned tomatoes contributed to the estimated daily intake of BPA (Table 4). The total daily intake of BPA in the selected foods commonly consumed in Southwest Nigeria by sex was 21.8 and 23.6 ng/kg bw/day for the males and females, respectively (Table 3). These values were lower than 35.1, 200–2000, 180 and 80 ng/kg bw/day reported in the USA^45^, Taiwan^37^, Canada^46^ and New Zealand^47^, respectively. The estimated daily intakes in the selected food categories by sex were subjected to T-test distribution to ascertain if there is a significant difference between them. There is no significant difference between the estimated daily intakes of BPA by males and females in Southwest Nigeria (p = 0.24 at α = 0.05).

**Table 3:** Estimated daily intake of BPA in selected foods commonly consumed by adults’ in Southwest Nigeria.

Food categories	Daily intake of BPA (ng/kg/day)	Daily intake of BPA (ng/kg/day)	
		Males	Females
Meat products	7.91	4.05	3.87
Aquatic foods	10.2	7.71	9.11
Dairy products	1.80	1.15	1.25
Edible oils	7.95	7.76	7.96
Fruits	0.49	0.00	0.00
Vegetables	2.04	0.24	0.26
Chicken eggs	0.00	0.90	1.14
Cereals	0.00	0.00	0.00
Total	30.4	21.8	23.6

**Table 4:** Estimated Daily intake of BPA in selected foods commonly consumed by adults in Southwest Nigeria by food types.

Food categories	Food types	Daily intake of BPA (ng/kg/day)
Meat products	Raw beef	0.00
	Raw chicken	0.00
	Canned beef	2.34
	Canned chicken	0.54
Aquatic food	Frozen fish	9.35
	Dried fish	9.58
	Canned fish	3.43
	Crayfish	2.69
Dairy products	Raw cheese	0.00
	Processed cheese	0.18
	Evaporated milk	3.21
Edible oils	Vegetable oils	8.19
	Palm oils	7.52
Chicken eggs	Eggs	0.00
Fruits	Raw apples	0.00
	Apple juice	0.87
Vegetables	Raw tomatoes	0.00
	Canned tomatoes	1.70
Cereals	Beans	0.00
	Rice	0.00

The values of HI of BPA in the selected foods commonly consumed in Southwest Nigeria ranged from 0.00–2.04 × 10^−4^ (Table 5). In all the food categories and food types, HI was lower than 1, which suggested no human risk of exposure to BPA by ingestion of the selected foods commonly consumed in Southwest Nigeria. HI > 1 indicates potential risk of human exposure. In most cases, the HI of all the selected food categories was higher in the females than in the males except in meat products (Table 5). Chicken eggs and cereals did not contribute to the health risk of BPA in both males and females in Southwest Nigeria. The HI obtained was subjected to T-test distribution to ascertain if there is a significant difference between the estimated health risk indices associated with males and females exposure to BPA in foods commonly consumed in Southwest Nigeria. There is no significant difference between them at α = 0.05 (p = 0.25).

**Table 5:** Health risk index (HI) of BPA in selected foods commonly consumed by adults’ in Southwest Nigeria.

Food categories	Health risk index (HI) of BPA	Health risk index (HI)	
		Males	Females
Meat products	1.58E-04	1.35E-03	1.29E-03
Aquatic foods	2.04E-04	2.57E-03	3.04E-03
Dairy products	3.60E-05	3.83E-04	4.17E-04
Edible oils	1.59E-04	2.59E-03	2.65E-03
Chicken eggs	0.00E + 00	0.00E + 00	0.00E + 00
Fruits	9.85E-06	7.88E-05	8.61E-05
Vegetables	4.08E-05	2.98E-04	3.80E-04
Cereals	0.00E + 00	0.00E + 00	0.00E + 00

## Discussion

Bisphenol-A concentrations in the selected food samples considered in this study were within the 600 ng/g, which is the European Union acceptable limit in food^41^. The results indicates the presence of BPA in some of the food categories. Exposure of humans to BPA primarily occurs through the hydrolysis of epoxy resins and polycarbonate plastics. This results in the release of low concentrations of free BPA into foods^48^. It also bio-accumulates in the aquatic animals as it gets into the environment through wastewater and sewage treatment discharges, and landfill leachates; and natural degradation of polycarbonate plastics^49^. Hence, the highest concentration was obtained in aquatic foods category in this study. Thus, food consumption is a known route of human exposure to BPA^50^.

Median concentrations of BPA in foods studied were differently compared with studies reported in other countries. The median concentrations of BPA in canned meat (11.9 ng/g) in this study was lower than the concentration reported in canned meat (98 ng/g) in New Zealand^47^ but slightly higher than the concentration (11 ng/g) obtained in canned meat in the United Kingdom^51^. Median concentration of BPA in canned fish (8.41 ng/g) obtained in this study was lower than what was reported in New Zealand^47^ (109 ng/g) and Iran (32.6 ng/g)^52^. The median concentration of BPA in canned tomato paste (1.38 ng/g) obtained in this study was slightly higher than the concentration reported (1.23 ng/g) in canned tomato paste in Iran^52^ but lower than the concentration reported (916 ng/g) in canned tomato paste in Nigeria^30^. The concentrations of BPA in the present study was also compared with what was reported in the United States^53^, where it was reported that BPA concentrations in meat and meat products, fish and seafood products, dairy products, fats and oils, and vegetable samples were 0.85, 3.23, 2.55, 1.90 and 8.99 ng/g, respectively. These were similar to what was obtained in this study. The health risk associated with BPA exposure through the daily intake of the selected foods considered in this study was within the permissible limit that will not pose any health risk to adults’ population in the study areas. Though, accumulation over time might be of concern. Routine monitoring of different foods commonly consumed in Nigeria is recommended to prevent human exposure to this toxic and endocrine disrupting chemical.

## Methods

### Chemicals and standards

All reagents and consumables used were of high purity (99.99%) and analytical grade. Acetonitrile and *n*-hexane were obtained from Merck (Darmstadt, Germany), anhydrous magnesium sulfate and sodium chloride were obtained from BDH Laboratories (UK) as reported elsewhere^43^. Reference standard of BPA was obtained from Sigma-Aldrich (St Louis, MO).

### Sample collection

Sample collection considered in this study was reported elsewhere^43^. Eight different food categories such as meat products, aquatic foods, dairy products, edible oil, eggs, fruits, vegetables, and cereals were selected and collected from 3 local markets in Mile-12, Ile-Epo and Bodija; and 2 superstores in Maryland and Dugbe in Lagos and Ibadan, Southwest Nigeria. A total of two hundred and forty-eight samples were collected between 2016 and 2017 as described elsewhere^43^. Briefly, three different samples were collected for each category at three different locations within the study areas and pooled together to form a composite. Raw chicken, beef, cheese, and eggs; and frozen fish were boiled and homogenised prior to extraction. Extract clean-up was achieved using the QuEChERS extraction kit. Canned beef, fish and chicken; seafood, dried fish, processed cheese, canned evaporated milk, vegetable and palm oil; raw apples and tomatoes; canned apple juice and tomato paste; raw beans and rice were homogenised without boiling prior to extraction^43^.

### Extraction and clean-up

QuEChERS extraction kit (Supelco) (Bellefonte, PA) was used for the extraction and clean-up^43,54,55^. The extraction and clean-up was reported elsewhere^43^. In summary, approximately 5 g each of the homogenised food samples were placed in 20 mL centrifuge tubes; 10 mL of acetonitrile was added and then shaken for 1 min after which 1 gram of NaCl and 4 g of MgSO_4_ were added and the tubes were shaken for 3 min. Samples were centrifuged for 5 min at 3400 rpm, and 1 mL each of the extracts (top layer) was transferred into a Supel™ QuE PSA/C18/ENVICarb (AC) tubes, shaken for 1 min and centrifuged for another 3 min at 3400 rpm.

### Derivatisation

Derivatisation was important for the targeted compound because of its low volatility. This aids the solubility of the analytes for detection by gas chromatography. Derivatisation was done using the procedure reported by^56^. One mL each of the extracts were transferred into 10 mL centrifuge tubes, 20 μL of 5% K_2_CO_3_ solution, 100 μL of tetrachloroethylene, 250 μL of acetic anhydride and 4 mL of distilled water were added. The tubes were sealed, shaken for 1 min and centrifuged at 3000 rpm for 3 min. 50 μL of the organic phase was transferred into the vials and made up to 1000 μL. One μL of the extracts were injected into GC–MSD.

### Instrumental analysis

Agilent (Santa Clara, CA) model 6890A gas chromatograph equipped with mass spectrometry detector (MSD) was used for sample identification and quantification. Column used was Agilent HP-5 ms (30 m (length) × 250 mm (internal diameter) × 0.25 μm (film thickness)) (J&W Scientific Inc. Folsom, CA). Instrument operating condition was: injector temperature, 250 °C; detector temperature, 250 °C; oven temperature was initially at 100 °C (0.5 min) and increased to 280 °C at 30 °C/min (5 min hold) with total run time of 12 min. Helium was used as the carrier gas with flow rate of 1 mL/min. Injection volume was 1 µL. BPA working standard at different concentrations: 2000, 1000, 500, 250, 125, 62.5, and 31.3 ng/mL were prepared for the calibration curve. Procedural blank was carried out; one blank for every ten samples was considered. The limit of detection and quantification were calculated based on the standard deviation of the response(s) and slope of the calibration curve (S) using Eqs 1 and 2, respectively^43^.

1 $${\rm{LOD}}=3.3\times (s/S)$$

2 $${\rm{LOQ}}=10\times (s/S)$$

where, LOD = limit of detection; LOQ = limit of quantification.

Previously analysed samples were spiked with BPA standard for the recovery study. BPA recoveries were determined by comparing the concentrations before and after spiking. % recovery was calculated using Eq. 3 ^43^:

3 $$\begin{array}{c}{\rm{ \% }}\,Recovery=Concentrations\,of\,BPA\,in\,spiked\,samples\\ /Concentrations\,of\,BPA\,in\,unspiked\,samples/Concentration\,of\,BPA\,standard\,added\end{array}$$

The recoveries ranged from 80 to 99%. The LOD was 0.00013 ng/g while LOQ was 0.0004. Linearity (*r2*) of the calibration curve was 0.998.

### Statistical analysis

The data obtained were subjected to statistical analysis such as mean, standard deviation, median, minimum, maximum, ANOVA and Duncan post-hoc test, and T-test distribution using the Statistical Package for the Social Sciences (SPSS) software 17.0 at *p* < 0.01.

### Human health risk assessment

#### Food consumption survey

Self-administered questionnaires were used for the food consumption survey in Lagos and Ibadan, Nigeria between 2016 and 2017 (Supplementary Table S4). The reference was a year prior to the year of the interview (study) (i.e., 2015 for those interviewed in 2016 and 2016 for those interviewed in 2017). The male and female adults were selected through composite sampling methods. The first step of the sampling involved stratified probability by Local Government Areas (LGAs). A total of 10 LGAs were randomly selected in Lagos and Ibadan. In the second step, 40 households were selected randomly from each LGAs, making a total of 400 households recruited for the study. Healthy looking adults with no form of physical deformity and sickness were recruited for the study. Informed consent was obtained from all the participants. No information about the identity of the participants was recorded during the survey and all the information obtained were treated in confidence. Information on the respondents’ sex, age, body weight, height, ethnicity, marital status and level of education were obtained from the questionnaires administered.

#### Estimated daily intake and health risk index (HI)

Data on dietary exposure of BPA in selected foods commonly consumed in Southwest Nigeria was obtained from the questionnaires administered to 250 adults’ respondents using a food frequency questionnaire (FFQ). The questionnaires consist of the sources of food, frequency of consumption (number of times per day, week, month or year) and the estimated quantity of food consumed. Daily intake (in g) of the selected foods was computed for the questionnaire (Supplementary Table S4). The FFQ was analysed for the dietary exposure of BPA via the consumption of the selected eight different food categories namely, meat products, aquatic foods, dairy products, edible oils, eggs, vegetables, fruits and cereals. The daily intake of BPA from the selected foods was estimated using Eq. 4 ^57^ while the health risk index was estimated using Eq. 5:

4 $$DI=Concentrations\,of\,BPA\,in\,food\times Average\,food\,intake/Average\,Body\,weight$$

5 $$HI=DI/RfD$$

where DI is the estimated daily intake of BPA, RfD is the reference dose (4,000 µg/kg-day), HI is the health risk index.

All methods were performed in accordance with the standard analytical procedures and guidelines.

### Ethical approval

This article does not contain any studies with human or animal subjects performed by any of the authors.

### Informed consent

Informed consent was obtained from all individual participants interviewed.

## Supplementary information

Supplementary Tables

## Data Availability

All data generated during the study were included in this published article (and its Supplementary Information file).
