# Supplementary material for: Bisphenol-A (BPA) in Foods commonly consumed in Southwest Nigeria and its Human Health Risk
Source: Sci Rep. 2019 Nov 25;9:17458. doi: 10.1038/s41598-019-53790-2 (PMC6877615; doi:10.1038/s41598-019-53790-2)
Supplement: Supplementary file 1 — Supplementary Tables [file 41598_2019_53790_MOESM1_ESM.pdf]

**Bisphenol-A (BPA) in Foods commonly consumed in Southwest Nigeria and its Human Health Risk**

A.A. Adeyi[a,b*] and B.A. Babalola[a,b]

[a]Department of Chemistry, University of Ibadan, Ibadan, Oyo State, Nigeria

[b]Basel Convention Coordinating Centre for Training and Technology Transfer for Africa Region, University of Ibadan, Ibadan, Oyo State, Nigeria

*Corresponding author e-mail address: bolaoketola@yahoo.com; Telephone Number: +234 8037763961.

Adebola A. Adeyi: ORCID: 0000-0002-6165-7458; LinkedIn: AdebolaAdeyi nee Oketola; Facebook: AdebolaOketola; Twitter: @bolaoketola

## Supplementary material

**Supplementary Table S1** **Mean concentrations (ng/g) of BPA in selected foods commonly consumed by adults' in Southwest Nigeria**

| Food categories | Food types | Total number of samples (n) | BPA concentrations (ng/g) |
|---|---|---|---|
| Meat products | Raw beef | 18 | ND |
| | Raw chicken | 24 | ND |
| | Canned beef | 4 | 12.7 |
| | Canned chicken | 4 | 4.42 |
| | **Mean** | | **4.28** |
| | **Stdev** | | **6.0** |
| Aquatic foods | Frozen fish | 18 | 4.06 |
| | Dried fish | 18 | 6.26 |
| | Canned fish | 4 | 11.2 |
| | Crayfish | 24 | 8.72 |
| | **Mean** | | **7.56** |
| | **Stdev** | | **3.1** |
| Dairy products | Raw Cheese | 4 | ND |
| | Processed cheese | 4 | 2.37 |
| | Evaporated milk | 4 | 1.89 |
| | **Mean** | | **1.42** |
| | **Stdev** | | **1.3** |
| Edible oil | Vegetable oils | 22 | 6.67 |
| | Palm oils | 18 | 6.11 |
| | **Mean** | | **6.39** |
| | **Stdev** | | **0.4** |
| Chicken eggs | Chicken eggs | 18 | ND |
| Fruits | Raw Apples | 12 | ND |
| | Processed apple juice | 4 | 0.40 |
| | **Mean** | | **0.20** |
| | **Stdev** | | **0.3** |
| Vegetables | Raw Tomatoes | 12 | ND |
| | Canned tomatoes | 4 | 2.21 |

| | | | |
|---|---|---|---|
| | **Mean** | | **1.11** |
| | **Stdev** | | **1.6** |
| Cereals | Beans | 18 | ND |
| | Rice | 18 | ND |
| | **Mean** | | **-** |
| | **Stdev** | | **-** |

**Supplementary Table S2** **Analysis of variance (ANOVA) of BPA in selected foods**

**ANOVA**

BPA

| | Sum of Squares | df | Mean Square | F | Sig. |
|---|---|---|---|---|---|
| Between Groups | 1585.870 | 19 | 83.467 | 4.369 | .000 |
| Within Groups | 1681.251 | 88 | 19.105 | | |
| Total | 3267.122 | 107 | | | |

**Supplementary Table S3** **Post-hoc homogenous subset (Duncan) of BPA in selected foods**

Duncan

| | | Subset for alpha = 0.05 | | | |
|---|---|---|---|---|---|
| Food samples | N | 1 | 2 | 3 | 4 |
| Beef | 6 | .0000 | | | |
| Chicken | 8 | .0000 | | | |
| Raw cheese | 4 | .0000 | | | |
| Chicken egg | 6 | .0000 | | | |
| Raw apple | 4 | .0000 | | | |
| Raw tomato | 4 | .0000 | | | |
| Beans | 6 | .0000 | | | |
| Rice | 6 | .0000 | | | |
| Processed apple juice | 4 | .4025 | | | |
| Evaporated milk | 4 | 1.8935 | | | |
| Canned tomato paste | 4 | 2.2120 | | | |
| Processed cheese | 4 | 2.3670 | | | |
| Frozen fish | 6 | 4.0577 | 4.0577 | | |
| Canned chicken | 4 | 4.4230 | 4.4230 | | |
| Palm oil | 6 | 6.1073 | 6.1073 | 6.1073 | |
| Dried fish | 6 | 6.2573 | 6.2573 | 6.2573 | |
| Vegetable oil | 10 | 6.6666 | 6.6666 | 6.6666 | |
| Crayfish | 8 | | 8.7205 | 8.7205 | 8.7205 |
| Canned fish | 4 | | | 11.1975 | 11.1975 |
| Canned beef | 4 | | | | 12.7122 |
| Sig. | | .052 | .146 | .105 | .178 |

Means for groups in homogeneous subsets are displayed.

**Supplementary Table S4** **Questionnaire administered**

**DEPARTMENT OF CHEMISTRY**
**UNIVERSITY OF IBADAN, IBADAN, NIGERIA**

Dear Respondent,

This questionnaire is designed purposely to collect information on consumption of certain food items with a focus to assess and ascertain the dietary pattern of these foods. Kindly give your response to each item as objectively as possible. Give estimate if you are not sure, a guess is better than leaving it blank. Please tick the correct answer or fill in the space provided.Your sincere response will immensely contribute to the success of this study. Your utmost confidentiality is, of course, guarantee since the information you provide will be used only for research purpose.

Thank you for your cooperation and contribution to this study.

**SECTION A:** Demographic information

Date of interview:
Place of interview:

**Personal data:**
Age:
16- 25 26 – 35 36 – 45 46 – 55 56 – 65 ≥66

Sex:
Male Female

Body weight:
Specify ____________________ kg

Height:
Specify____________________ cm

Ethnicity:
Specify____________________

Marital status:
Single Married Separated Widow

Level of highest education:
No formal education Primary school Secondary school
Tertiary education

**SECTION B: Food consumption frequency**

This questionnaire is designed to find out how often you eat particular types of food. Please answer as accurately as possible. Do not leave any blank questions. For each item of food, please indicate how many times you eat it by ticking the appropriate number. Thank you for your cooperation and time.

| Food Samples | Sources of food | |
|---|---|---|
| | Local markets | Supermarkets |
| A. Meat products | | |
| Raw beef | | |
| Raw chicken | | |
| Canned beef | | |
| Canned chicken | | |
| | | |
| B. Aquatic foods | | |
| Frozen fish | | |
| Dried fish | | |
| Crayfish | | |
| Canned fish | | |
| | | |
| C. Dairy products | | |
| Raw cheese | | |
| Processed cheese | | |
| Evaporated milk | | |
| | | |
| D. Edible oil | | |
| Vegetable oil | | |
| Palm oil | | |
| | | |
| E. chicken eggs | | |
| | | |
| F. Vegetables | | |
| Raw tomatoes | | |
| Canned tomatoes | | |
| | | |
| G. Fruits | | |
| Raw fruits (apple) | | |
| Canned juice (apple) | | |
| | | |
| H. Cereals | | |
| Beans | | |
| Rice | | |

| Food samples | Frequency of consumption of food items | | | | | | | Quantity consumed | | | |
|---|---|---|---|---|---|---|---|---|---|---|---|
| | Never | <Once per month | 1-3 times per month | once per week | 2-4 times per week | Once per day | 2-3 times per day | Don't know | Per month | Per week | Per day |
| Meat products | | | | | | | | | | | |
| Beef (g) | | | | | | | | | | | |
| Chicken (g) | | | | | | | | | | | |
| Canned beef (g) | | | | | | | | | | | |
| Canned chicken(g) | | | | | | | | | | | |
| | | | | | | | | | | | |
| Aquatic food | | | | | | | | | | | |
| Frozen fish (g) | | | | | | | | | | | |
| Dried fish (g) | | | | | | | | | | | |
| Crayfish (g) | | | | | | | | | | | |
| Canned fish (g) | | | | | | | | | | | |
| | | | | | | | | | | | |
| Dairy products | | | | | | | | | | | |
| Cheese (g) | | | | | | | | | | | |
| Processed cheese (g) | | | | | | | | | | | |
| Evaporated milk (mL) | | | | | | | | | | | |
| | | | | | | | | | | | |
| Edible oil | | | | | | | | | | | |
| Vegetable oil (mL) | | | | | | | | | | | |
| Palm oil (mL) | | | | | | | | | | | |
| | | | | | | | | | | | |
| Eggs | | | | | | | | | | | |
| Fruits | | | | | | | | | | | |
| Raw fruits (apple) (g) | | | | | | | | | | | |
| Canned juice (apple) (mL) | | | | | | | | | | | |
| | | | | | | | | | | | |
| Vegetables | | | | | | | | | | | |
| Tomato (g) | | | | | | | | | | | |
| Canned tomato (g) | | | | | | | | | | | |
| | | | | | | | | | | | |
| Cereals | | | | | | | | | | | |
| Beans (g) | | | | | | | | | | | |
| Rice (g) | | | | | | | | | | | |

**Supplementary Table S5 Characteristics of the respondents**

| Demographic information /sex | | Males | Females | % Respondents |
|---|---|---|---|---|
| Total number of respondents that participated in the study | 250 | 100 | 150 | |
| | | | | |
| Age | 16-25 | Nil | Nil | |
| | 26-35 | 18 | 27 | 18 |
| | 36-45 | 21 | 30 | 20.4 |
| | 46-55 | 25 | 35 | 24 |
| | 56-65 | 24 | 38 | 24.8 |
| | >65 | 12 | 20 | 12.8 |
| | | | | |
| Average body weight | | 63 kg | 67 kg | |
| Average height | | 1.55 m | 1.32 m | |
| Ethnicity | | Yoruba | Yoruba (125) and Igbo (25) | |
| Marital status | Single | Nil | Nil | |
| | Married | 100 | 140 | |
| | Separated | Nil | 8 | |
| | Widow | Nil | 2 | |
| Levels of education | No formal education | Nil | Nil | |
| | Primary education | Nil | Nil | |
| | Secondary education | 10 | 37 | |
| | Tertiary education | 90 | 113 | |
